# The role of microglial cells in amyloid propagation and aggregation

**DOI:** 10.1002/alz.092512

**Published:** 2025-01-03

**Authors:** Juana Andreo‐Lopez, Miriam Bettinetti‐Luque, Kelly Do Huynh, Marie Minh Thu Nguyen, Alwin Cheung, Janine Pham Tran, Celia Da Cunha, Laura Trujillo‐Estrada, Cynthia Campos‐Moreno, Juan Antonio Garcia Leon, Ivan Fatuarte‐Juli, Mario Morales‐Cabello, Cristina Nuñez‐Diaz, Alessandra Cadete Martini, Stefania Forner, Antonia Gutierrez, Frank M LaFerla, David Baglietto‐Vargas

**Affiliations:** ^1^ University of Malaga/CIBERNED/IBIMA, Málaga Spain; ^2^ University of California, Irvine, Irvine, CA USA

## Abstract

**Background:**

Alzheimer’s Disease (AD) is a neurodegenerative proteinopathy in which Aβ can misfold and aggregate into seeds that structurally corrupt native proteins, mimicking a prion‐like process. These amyloid aggregation and propagation processes are influenced by three factors: the origin of the Aβ seed, time of incubation and host. However, the mechanism underlying the differential effect of each factor is poorly known. Previous studies have shown that the Aβ source is relevant for the amyloid process, since its pathogenicity is different according to its origin. Furthermore, recent evidence suggests that microglia plays a key role in the amyloidogenic event, and can modulate the propagation and aggregation process. Here, we seek to perform a comparative study to determine whether Aβ seeds from humans vs a familial AD line (the 3xTg‐AD model) are more efficient to generate amyloid aggregates, as well as the role of the microglia in the propagation process.

**Method:**

Amyloid seeds from AD patient (stage C for amyloid; from the Alzheimer’s Disease Research Center at UCI) and 25 mo‐3xTg‐AD mice were injected into the hippocampus of 7‐8‐month‐old 3xTg‐AD mice. They were analyzed 10 months post‐surgery for amyloid and microglia markers.

**Results:**

Our findings demonstrated that amyloid seeds from the human patient seem to induce a more aggressive amyloid pathology compared to seeds from aged 3xTg‐AD mice. Moreover, human and mice seeds differentially affect the presence of plaque‐associated microglia in 3xTg‐AD mice.

**Conclusion:**

These results suggest that seeds from human patients seem to be more amyloidogenic than from aged 3xTg‐AD mice, and also microglia cells may play a key role in this differential effect. Therefore, more profound understanding these factors will provide key insight on how amyloid pathology progresses in AD.

**Funding**: This study was supported by Minister of Science and Innovation grant PID2019‐108911RA‐100 (D.B.V.), Alzheimer’s Association grant AARG‐22‐928219 (D.B.V), Beatriz Galindo program BAGAL18/00052 (D.B.V.) and Institute of Health Carlos III (ISCiii) grant PI18/01557 (A.G.) co‐financed by FEDER funds from European Union.

